# Prognostic Implication of Plasma Metabolites in Gastric Cancer

**DOI:** 10.3390/ijms241612774

**Published:** 2023-08-14

**Authors:** Kang Cao, Yanping Lyu, Jingwen Chen, Chenzhou He, Xuejie Lyu, Yuling Zhang, Liangping Chen, Yu Jiang, Jianjun Xiang, Baoying Liu, Chuancheng Wu

**Affiliations:** 1 Department of Preventive Medicine, School of Public Health, Fujian Medical University, Fuzhou 350122, China; flechazo@fjmu.edu.cn (K.C.);; 2 The Key Laboratory of Environment and Health, School of Public Health, Fujian Medical University, Fuzhou 350122, China

**Keywords:** gastric cancer, prognosis, metabolites, metabolic signatures

## Abstract

Gastric cancer (GC) typically carries a poor prognosis as it is often diagnosed at a late stage. Altered metabolism has been found to impact cancer outcomes and affect patients’ quality of life, and the role of metabolites in gastric cancer prognosis has not been sufficiently understood. We aimed to establish a prognostic prediction model for GC patients based on a metabolism-associated signature and identify the unique role of metabolites in the prognosis of GC. Thus, we conducted untargeted metabolomics to detect the plasma metabolites of 218 patients with gastric adenocarcinoma and explored the metabolites related to the survival of patients with gastric cancer. Firstly, we divided patients into two groups based on the cutoff value of the abundance of each of the 60 metabolites and compared the differences using Kaplan–Meier (K-M) survival analysis. As a result, 23 metabolites associated with gastric cancer survival were identified. To establish a risk score model, we performed LASSO regression and Cox regression analysis on the 60 metabolites and identified 8 metabolites as an independent prognostic factor. Furthermore, a nomogram incorporating clinical parameters and the metabolic signature was constructed to help individualize outcome predictions. The results of the ROC curve and nomogram plot showed good predictive performance of metabolic risk features. Finally, we performed pathway analysis on the 24 metabolites identified in the two parts, and the results indicated that purine metabolism and arachidonic acid metabolism play important roles in gastric cancer prognosis. Our study highlights the important role of metabolites in the progression of gastric cancer and newly identified metabolites could be potential biomarkers or therapeutic targets for gastric cancer patients.

## 1. Introduction

Gastric cancer (GC) is one of the most prevalent causes of cancer-related deaths worldwide [1]. Over 95% of gastric cancers are adenocarcinomas, which are typically classified based on anatomic location and histologic type [2]. Despite significant advancements in the diagnosis and treatment of gastric cancer over the past few decades, the overall survival rate of patients with this disease remains unsatisfactory [3]. The main treatment for early gastric cancer is endoscopic resection, while chemotherapy is mainly used for advanced gastric cancer with a median survival period of less than 1 year and a poor prognosis [1]. The main factors affecting the prognosis of gastric cancer include age, Tumor Node Metastasis (TNM) clinical stage, depth of infiltration, positive lymph nodes, tumor differentiation degree, tumor diameter, etc. Although prognosis judgments in medicine have made significant strides, relying primarily on personal medical experience still prevails. Unfortunately, such means of judgment present limitations due to the lack of efficient, accurate, and reliable prognostic markers available. Targeted therapies have produced encouraging results in the treatment of patients with advanced gastric cancer [4]. However, cancer progression, immune responses, inflammation, and neurological changes remain significant barriers to developing targeted therapies. Consequently, it is crucial to actively search for these biomarkers to predict disease progression accurately as well as possible therapeutic effects. A reliable prognosis marker would guide clinical decisions efficiently and help healthcare experts improve the survival time and quality of life of cancer patients, making the quest for these biomarkers a valuable endeavor.

Metabolic reprogramming is a major hallmark of cancer, as cancer cells independently alter multiple metabolic pathways to promote survival and proliferation. There is growing evidence that reprogrammed cellular metabolism supports tumorigenesis, progression, metastasis, and drug resistance [5,6]. With advances in modern science, there is a growing focus on metabolism and tumor biology. Transformed cells adapt their metabolism to support the biological process of neoplasia. Specific metabolic pathways directly participate in transformation and tumor progression. The blocking of these pathways or restoration of altered metabolic pathways has been proven to be promising therapeutic target strategies [7,8]. Metabolomics seeks to capitalize on the metabolic signature of cancer to assess disease risk or for earlier cancer detection, diagnosis of specific disease subsets, or treatment monitoring. It provides valuable information on the metabolic profile of specific tumors and identifies key biomarkers for cancer diagnosis and prognosis [9,10]. Metabolomics, in principle, may also help inform the rational selection of targeted therapies to match the metabolic dependencies of cancer. This enables the development of precision medicine approaches for cancer treatment [11,12].

Tumor metabolic reprogramming includes upregulated aerobic glycolysis, increased glutamine, and lipid accumulation, which support cancer cell development [13]. Alterations to metabolic pathways can affect the survival of patients with gastric cancer. For example, tissue-resident memory T (Trm) cells are frequent in the tumor environment, which has been linked to better outcomes in patients with gastric adenocarcinoma. Trm cells mainly depend on fatty acid oxidation, but deprivation of fatty acids by cancer cells can lead to Trm death [14]. Targeted programmed cell death-ligand 1(PD-L1) can promote lipid uptake by Trm cells, increase their survival rate, improve patient outcomes, and have promoted the mode transformation of cancer treatment [15]. In general, the treatment of GC has entered the stage of personalized precision treatment. At present, there are many drugs and combination modes of immunotherapy for GC. Selecting appropriate biomarkers for screening suitable patients and monitoring therapeutic efficacy is the first step toward the success of immunotherapy. The combined application of immune drugs and targeted drugs in chemotherapy and adjuvant chemotherapy is the current strategy and the future research direction of immunotherapy for GC [16].

Metabolomics can support research into predicting metastasis, recurrence, and drug resistance in gastric cancer [17]. A few studies have revealed several significant metabolic alternations that are sensitive to GC. Huang et al. suggested that α-linolenic acid, linoleic acid, and palmitic acid may be meaningful biomarkers for assessing high-risk populations and early diagnosis of gastric cancer, possibly advancing targeted GC prevention and control [18]. An in-depth understanding of abnormal tumor metabolism can provide insights into the overall development of cancer and lead to the development of cancer metabolic drugs and therapies. Targeted metabolic modulation is expected to be a strong and effective treatment strategy for gastric cancer. This study aims to investigate the relationship between metabolites and gastric cancer prognosis using non-targeted metabolomics, which will provide a scientific basis for studying targeted metabolites and as a new concept for targeted regulation of metabolic therapy.

## 2. Results

### 2.1. Study Sample Characteristics

**Figure 1:**
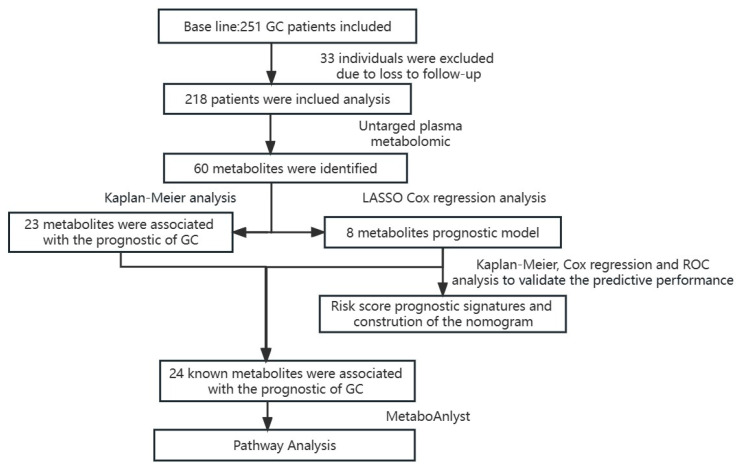
The flow chart summarizes the scheme performed to construct prognostic risk score signatures of gastric cancer.

We conducted our study as illustrated in the flow chart (Figure 1). A total of 218 GC cases were included in this follow-up study. The Kaplan–Meier (K-M) analysis and Cox regression for clinicopathological parameters, including tumor (T), node (N), metastasis (M), and overall stage, are shown in Table 1. The patient’s age, TNM stage, surgery, and chemotherapy were all associated with their prognosis (*p* < 0.05). Among them, age greater than or equal to 65 years or intermediate to advanced TNM stage were high-risk factors for poor prognosis, while surgery and chemotherapy reduced the risk of poor prognosis in gastric cancer.

### 2.2. Metabolic Profiles of Plasma Samples

**Figure 2:**
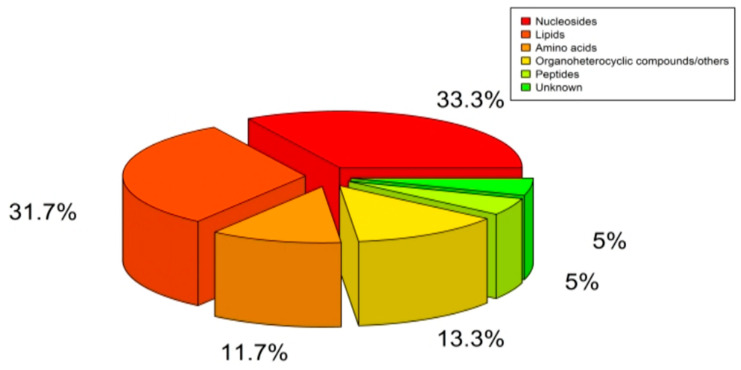
Classification of Detected Plasma Metabolites.

After being excluded based on Extracted Ion Chromatogram (EIC) and structurally identified by comparing with data shown in HMDB (www.hmdb.ca, accessed on 15 September 2021) and Thermo mzCloud databases, 60 compounds were identified from six chemical classes, of which 20 were nucleotides, 19 were lipids, 7 were amino acids, 3 were peptides, 8 were carbohydrates/other, and 3 were unknown metabolites (Figure 2).

### 2.3. The Association between Plasma Metabolites and the Prognosis of Gastric Cancer

**Figure 3:**
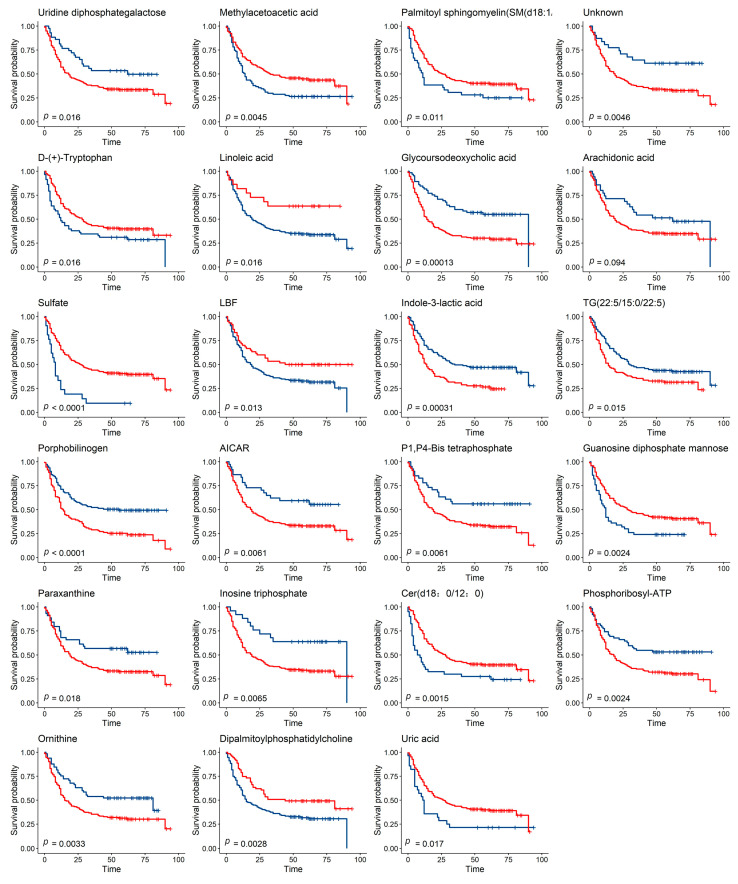
Metabolites Associated with the Survival of GC Patients. Two groups in the K-M survival analysis were divided according to the cutoff value of metabolites, and the survival time was compared using the log-rank test (the blue line represents group 1, and the red line represents group 2, FDR_*p* < 0.05).

Relative abundance values of plasma metabolites are continuous variables, and we used the surv_cutpoint from survminer package (R 4.0) to select suitable cutoff points. We then divided patients into two groups based on the cutoff value of the abundance of each of the 60 metabolites and compared the differences using Kaplan–Meier (K-M) survival analysis. Our results showed that 23 metabolites were associated with the prognosis of gastric cancer (Figure 3).

### 2.4. Prognosis Model of Gastric Cancer

**Figure 4:**
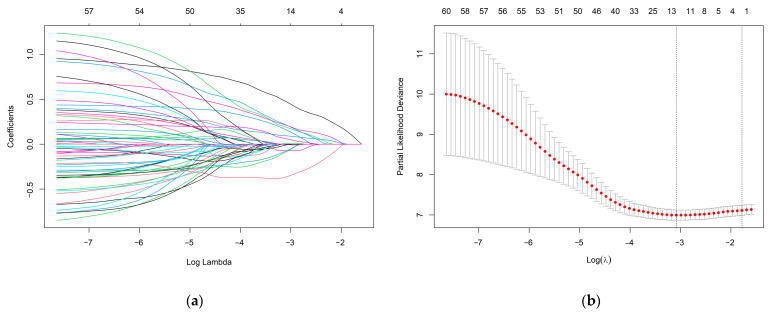
(**a**) LASSO coefficient profiles of the 60 metabolites. Different colors lines represent different metabolites. (**b**) A coefficient profile plot was produced against the log (lambda) sequence in the LASSO model. The red dots in the figure represent target parameters for each lambda value. In the graph, there are two dashed lines representing special lambda values. The first line on the left corresponds to the lambda value that minimizes the mean squared error, while the other line represents a lambda value that differs from it by one standard deviation. The optimal parameter (lambda) was selected as the first black dotted line indicated.

**Figure 5:**
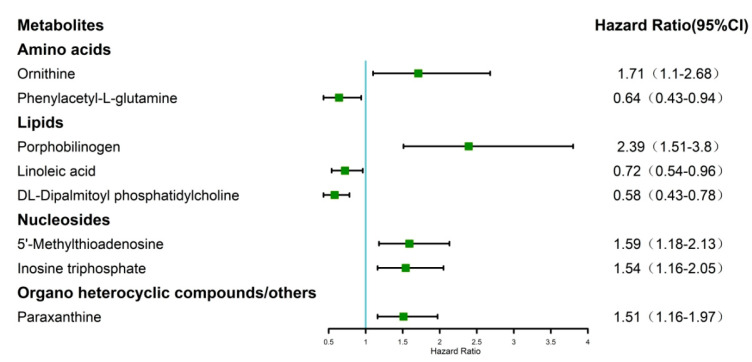
The forest plot of the Cox regression analysis indicated eight metabolites associated with the survival status of GC patients.

To identify the most valuable predictive metabolites for gastric cancer prognosis, we additionally screened variables using least absolute shrinkage and selection operator (LASSO) regression and constructed a Cox proportional hazards model. The optimal model, with good performance and the lowest number of independent variables, was selected through cross-validation. Results indicated that the best Lasso regression model screened 14 variables (Figure 4). Furthermore, 14 variables were selected for Cox regression, and 8 metabolites related to survival status were found to establish metabolic risk signatures (Figure 5).

**Figure 6:**
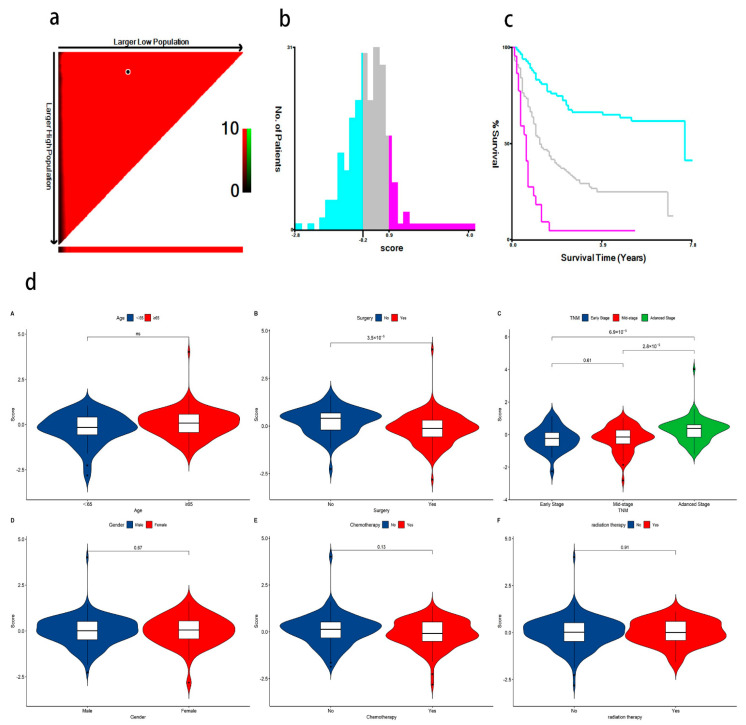
(**a**) The optimal cut-off points of risk scores identified by X-tile software (version 3.6, Yale University, New Haven, CT, USA); (**b**) GC patients were divided into three risk score levels (low, medium, and high) based on the cut-off value. The blue color represents low-risk for poor prognosis, gray represents medium-risk, and pink represents high-risk for poor prognosis; (**c**) K-M survival analysis shows a significant difference in survival time among the three groups (The blue line represents the low-risk group, the gray line represents the moderate-risk group, and the pink line represents the high-risk group. *p* < 0.05); (**d**) (**A**–**F**) Violin plots show that surgery and TNM stage are significant with the association of metabolite risk scores.

**Figure 7:**
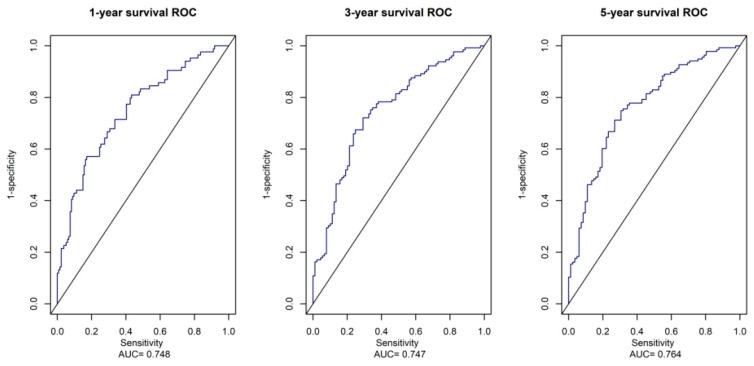
ROC Analysis of the Prognostic Metabolites Signature. The AUC of 1-year, 3-year, and 5-year survival curves show good sensitivity and specificity predictive performance of the metabolite risk scores.

**Figure 8:**
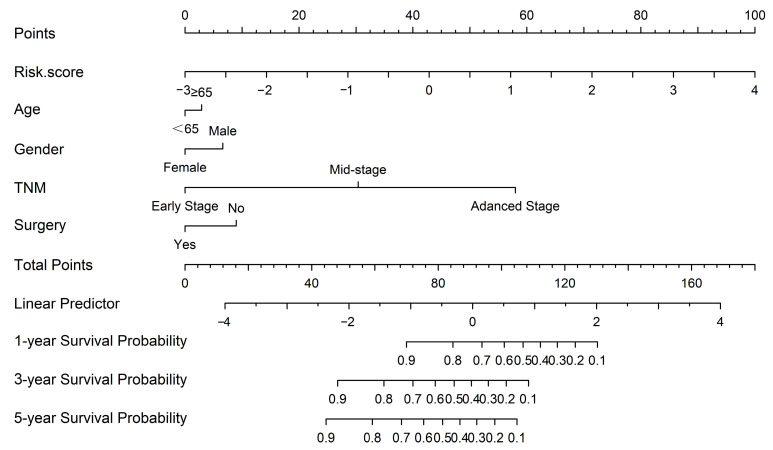
The nomogram was constructed based on five independent prognostic factors.

**Figure 9:**
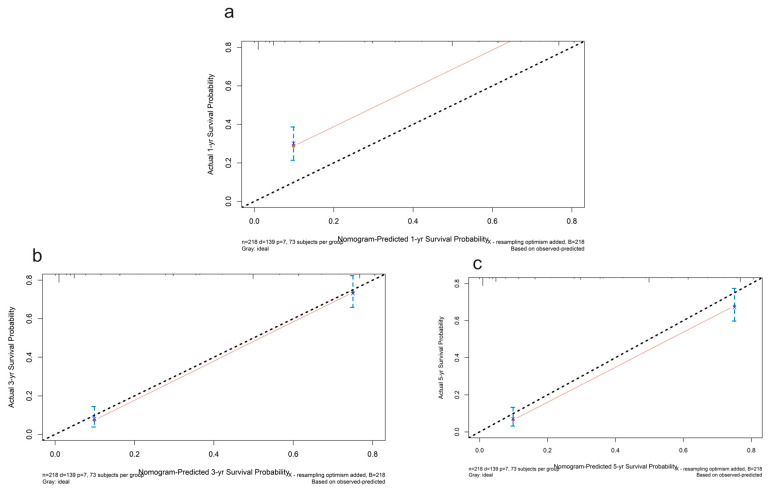
The calibration plots for the internal validation of the nomogram predicting 1-year (**a**), 3-year (**b**), and 5-year (**c**) survival probability. The y axis represents the nomogram actual survival, and the x axis represents the predicted survival. The diagonal dotted line represents a perfect prediction by an ideal model. The red line represents the performance of the nomogram. The blue bars indicate the confidence intervals.

Risk scores for each patient were calculated according to a formula and the best cut-off value was determined using X-tile software (version 3.6, Yale University, New Haven, CT, USA) (Figure 6a). X-tile data are displayed in a right-angled triangular grid, where each point represents a different cut point. The color intensity of each cut point represents the strength of the association. The optimal cut-off point is determined by locating the brightest pixel on the X-tile plot of the training set. The patients were then classified into three categories: low risk of poor prognosis, medium risk, and high risk (Figure 6b). There were 83 patients in the low-risk group, 113 patients in the middle-risk group, and 22 patients in the high-risk group. The results showed a significant difference in survival time among the three groups, indicating that this feature has good predictive ability (Figure 6c). In addition, the signatures were found to be associated with TNM staging and surgery in GC patients. The predictive performance of the metabolic risk score was further assessed by plotting receiver operating characteristic curve (ROC) curves and the nomogram diagram. The area under the ROC curve (AUC) values were 74.8%, 74.7%, and 76.4% at 1, 3, and 5 years (Figure 7). The nomogram is an efficient tool that integrates multiple risk factors for clinical application. We established a nomogram for the prediction of survival probability in GC patients, and the C-index of our nomogram was 0.834 (95% CI = 0.804–0.864). Five independent risk factors, including age, gender, TNM stage, surgery, and metabolism signature, were included in the model (Figure 8), respectively, and the calibration curves showed that the predicted survival of the prognostic model fitted well with actual survival (Figure 9). The results suggest that our prognosis prediction model has good sensitivity and specificity, and the predictive performance is satisfied.

### 2.5. Survival-Related Metabolite Pathway Analysis

**Figure 10:**
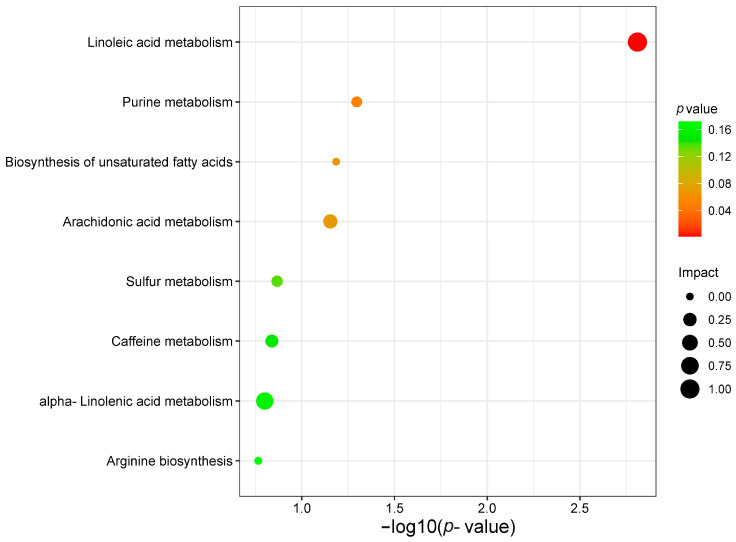
Analysis of gastric cancer survival-related metabolite pathways.

A total of 24 known metabolites were identified as being associated with survival in gastric cancer. Subsequent pathway analysis was to reveal how these metabolites influence prognosis in GC. In this study, we included 24 metabolites in the MetaboAnalyst (https://www.metaboanalyst.ca, accessed on 10 November 2021) platform for enrichment analysis and pathway analysis. The major pathway of metabolites associated with gastric cancer survival was purine metabolism and linoleic acid metabolism (Figure 10).

## 3. Discussion

Gastric cancer is a malignant tumor with a poor prognosis, and there are currently no effective methods to cure this disease. In recent years, targeting cancer metabolism with precision has been considered highly effective in the clinic [19]. It will be meaningful to find potential therapeutic targets in the common metabolic pathway [20]. To our knowledge, prognostic metabolic signatures and the role of metabolites and metabolic pathways in GC have not yet been well understood. In this study, we explored metabolic molecules that play a role in gastric cancer prognosis using untargeted metabolomics, and two statistical methods were used to screen for novel metabolites that may be associated with the survival of GC patients.

We plotted ROC curves for the metabolic risk features established based on LASSO and COX regression analysis, of which, the AUC was 0.748, 0.747, and 0.764. In addition, based on metabolic risk signatures and clinicopathological parameters, we constructed a nomogram to construct and visualize a statistical predictive model. This nomogram enables the generation of a numerical probability for individual clinical outcomes. Owing to their intuitive visual presentation and personalized application, nomograms have become a popular tool for oncology prognosis [7,21]. Lu et al. [22] constructed a stomach adenocarcinoma prognostic signature based on anoikis-related lncRNAs and clinical significance. The values of AUC at 1, 3, and 5 years were 0.62, 0.69, and 0.68, respectively. Additionally, Di et al. [23] developed a novel nomogram integrated with PDL1 and CEA to predict the prognosis of patients with gastric cancer, and the C-index was 0.763 (95% CI 0.740–0.787). To our knowledge, our model parameters are superior to most of the prognostic models for gastric cancer, and we have identified specific metabolic features that play a crucial role in the prognosis of gastric cancer.

Our study contained a variety of metabolites, including lipids, nucleotide-based chemicals, amino acids, organic heterocyclic compounds, and peptide metabolites. We then analyzed the metabolic pathways affected by the 24 metabolites to deepen our understanding of the role of metabolites in gastric cancer prognosis. The identification of these metabolites and pathways has the potential to provide new insights into the underlying mechanisms of gastric cancer progression. Moreover, it may aid in developing novel prognostic biomarkers and therapeutic targets for this disease.

### 3.1. Lipid Metabolism

Lipid metabolism, particularly fatty acid synthesis, plays a crucial role in converting nutrients into metabolic small molecules that are required to produce synthetic membrane organisms, energy reserves, and signaling molecules [11]. Dysregulated lipid metabolism is a prominent metabolic alteration in cancer, and it can induce lipid uptake to adapt to alterations in the tumor microenvironment and benefit cellular processes associated with tumor progression. A variety of lipid metabolites in tumor cells can also be targeted for cancer therapy [24]. In this work, an association was found between nine lipid metabolites and survival in gastric cancer patients. Porphobilinogen, indole-3-lactic acid, glycoursodeoxycholic acid, TG(22:5/15:0/22:5), and arachidonic acid were associated with an increased risk of poor prognosis. Conversely, linoleic acid, SM(d18:1/16:0), DL-dipalmitoylphosphatidylcholine, and Cer(d18:0/12:0) were associated with a lower risk of poor prognosis.

Linoleic acid is the most abundant polyunsaturated fatty acid in human nutrition, and polyunsaturated fatty acids have some tumoricidal effects [25]. Linoleic acid inhibits the growth effect of gastric epithelial adenocarcinoma cells, which is consistent with the findings of the present study. While previous studies have found that the arachidonic acid metabolic pathway plays a pro-carcinogenic role in cancer development [26,27], it is significantly negatively associated with overall gastric cancer risk [18]. Its complex biological mechanisms in cancer need further functional experiments to be explored.

SM(d18:1/16:0), DL-dipalmitoylphosphatidylcholine, and Cer(d18:0/12:0) all belong to sphingolipids [28]. The synthesis and/or accumulation of ceramide in response to cellular stress mediates cancer cell death through various mechanisms of apoptosis, necroptosis, and/or mitochondrial autophagy, which can also affect cancer cell growth, drug resistance, and tumor metastasis. Sphingolipids, particularly gangliosides, appear to be ideal antigens to label indelible tumor cells that enable their recognition by immune cell effectors as therapeutic targets for cancer research [29,30,31]. The results here found an association between plasma sphingolipids and longer survival times in gastric cancer, suggesting that sphingolipid-related metabolism during gastric cancer progression could induce cancer cells towards extinction and may be a focus for future targeted therapies.

### 3.2. Nucleotide Metabolism

Nucleotides are key components of DNA and RNA structure, and impairments in their biosynthesis have profound implications for cell physiology and may lead to tumor transformation in cells [32]. Nucleotide metabolism is critical for the growth and proliferation of cancer cells, which depend on the de novo synthesis of nucleotides from glucose-derived ribose and the amino acids that produce purine and pyrimidine nucleotides. Abnormalities in nucleotide biosynthesis can have profound implications for cell physiology and may lead to tumor transformation. Currently, approved drugs target nucleotide metabolism in the clinic, but normal cells also rely on nucleotide synthesis for proliferation, and targeted therapies for cancer must achieve precise inhibition of cancer cell proliferation without harming normal cells [19].

In this context, we identified eight nucleotides associated with prognostic outcomes of gastric cancer patients. Inosine triphosphate, 5′-Methylthioadenosine, 5-Aminoimidazole-4-carboxamide nucleotide (AICAR), uridine diphosphate galactose, phosphoribosyl-ATP, and 5′-Uridyl tetraphosphate were associated with bad prognostic outcomes in patients with gastric cancer, while uric acid and guanosine diphosphate mannose were associated with relatively good prognostic outcomes in gastric cancer patients.

5′-Methylthioadenosine is a naturally occurring sulfur-containing nucleoside that has been found to inhibit tumors such as melanoma and colorectal cancer mainly by inhibiting tumor cell proliferation, invasion, inducing apoptosis, and controlling the inflammatory microenvironment of the tumor tissue [33,34,35]. AICAR is an analog of adenosine monophosphate and may inhibit tumors by switching off Warburg metabolism and turning on anti-Warburg metabolism through mitochondrial regulation. AICAR may also activate AMP-activated protein kinase (AMPK) to sensitize prostate cancer cells to radiotherapy [36,37].

Adenosine triphosphate (ATP) is one of the main biochemical components of the tumor microenvironment (TME) and can contribute to tumor progression or tumor suppression, depending on its concentration and the specific nucleases and receptors expressed by immune cells and cancer cells. ATP in the TME has a central role in determining tumor fate and is, therefore, a suitable target for cancer therapy [38]. Uric acid, the product of purine metabolism via xanthine oxidoreductase, has a dual effect on cancer. When uric acidemia is within the normal range, uric acid is thought to scavenge free radicals and contribute to the total antioxidant capacity of plasma [39].

### 3.3. Amino Acid Metabolism

Amino Acid Metabolism Abnormalities in amino acid metabolism are common in cancer, but the acquisition and utilization of amino acids varies between different cancers [40]. The study found three amino acid metabolites to play a role in gastric cancer patient survival, with ornithine promoting cancer progression, and phenylacety-L-glutamine and D-(+)-tryptophan inhibiting cancer progression.

Ornithine is a non-essential amino acid involved in the regulation of metabolic disorders, and its depletion is often observed in patients with gastric cancer with lymph nodes and distant metastases [41,42]. Our study found that ornithine promotes the progression of gastric cancer, implying that it may drive cancer cell proliferation and distant metastasis. Tryptophan metabolism produces degradation products that are known to promote cancer cell motility and impair the anti-tumor response of the immune system, and it is an important mechanism used by cancer to evade immune surveillance [43,44].

### 3.4. Other Metabolites

Our results also found paraxanthine to be a risk factor for poor prognosis in gastric cancer, while Pantetheine (LBF), methylacetoacetic acid, and sulfate were protective factors for poor prognosis in gastric cancer. Notably, a definitive answer to the role of xanthine in gastric cancer has not yet been obtained. However, it has consistently been found to be low in patients with advanced pancreatic cancer malignancy [45]. Additionally, it differs significantly in patients with advanced non-small cell lung cancer who have better and worse survival rates when receiving first-line chemotherapy [46]. The metabolism of cancer is influenced by a wide range of complex factors. Therefore, there may be some variation in different populations in different regions. Further research is needed to elucidate the exact role of these metabolites in gastric cancer prognosis and to determine their potential as therapeutic targets in the future.

An obstacle to targeting metabolism for cancer therapy is to determine which specific pathways cancer affects, further deeply resolving aberrant metabolic pathways, and inhibiting the progression of cancer through the understanding of metabolism to discover emerging cancer therapies that are widely studied [47,48]. Bioinformatics analysis can associate metabolites and their corresponding biological functions to understand cancer development and treatment through metabolomics application in cancer metabolic reprogramming [49,50].

In this study, the purine and linoleic acid metabolic pathways were found to be the most relevant metabolic pathways associated with the prognosis of gastric cancer. Purines are the most abundant metabolic substrates in all organisms and play essential roles in cell survival and proliferation. Dysregulation of purine metabolism is closely associated with cancer development, making it a potential therapeutic target [51]. Linoleic acid is widely involved in cancer development, progression, and metastasis, and a better understanding of its physiological functions may suggest improvements in dietary or pharmacological interventions for the treatment of gastric cancer. This finding suggests that targeting and regulating these metabolic pathways or developing targeted metabolic drugs could help halt the progression of cancer, and help develop potential solutions to overcome the challenges associated with cancer advancement.

## 4. Materials and Methods

### 4.1. Study Participants and Sample Collection Metabolite Profiling

This study was based on a population-based prospective cohort study, in which GC patients were consecutively enrolled in Xianyou County between March 2013 and December 2017. All newly diagnosed cases of GC which were confirmed histologically based on tissue specimens and who had lived in Xianyou for at least 10 years were included. GC patients with other cancers; secondary or recurrent gastric cancer; gastritis; who previously received neoadjuvant chemotherapy or chemoradiotherapy or radiotherapy; who were pregnant; with metabolic diseases such as diabetes, gout, and hyperlipidemia; systemic administration of corticosteroids; neurological and psychiatric diseases; severe hepatic and renal dysfunction; and severe respiratory disease requiring continuous oxygen treatment, etc., were excluded. A total of 218 GC cases were recruited. The current research was approved by the Biomedical Research Ethics Committee of Fujian Medical University, China (no. 97,2014), in line with the relevant regulations and requirements of medical ethics. Written consent was obtained from all participants at study enrollment. Participant recruitment, anthropometric measurements, blood sample collection, and other details can be found in the previous study [52,53]. Definition of survival time: from the date of diagnosis to the date of death (if the study subject died), or to the time of termination of the last follow-up visit (if still alive).

### 4.2. Metabolite Profiling Data Processing and Statistical Analysis

After thawing the plasma samples, 450 μL of acetonitrile was added to 150 μL of plasma, vortexed, and held at 4 °C for 3 h to fully precipitate the proteins. The mixture was centrifuged at 16,000 RCF for 30 min at 4 °C. Then, 150 μL of supernatant was transferred into LC-MS vials for further analyses.

Each sample was processed using Agilent 1200 HPLC coupled with a 6520-precision electrospray ionization/quadrupole time-of-flight mass spectrometry system (Agilent Technologies, Santa Clara, CA, USA). Plasma samples were separated on an Agilent SB-C18 column (2.1 × 50 mm, 1.8 μm, Agilent Technologies, Santa Clara, CA, USA) with an injection volume of 20 μL at a flow rate of 0.25 mL/min and a column temperature of 37 °C using a gradient program of 0.1% formic acid solution (ESI+)/water (ESI−) in mobile phase A and acetonitrile containing 0.1% formic acid solution (ESI+)/water (ESI−) in mobile phase B solution (ESI+)/acetonitrile (Merck, Darmstadt, Germany) (ESI−). The gradient program started at 2% B for 0–9 min, increased linearly from 2% B to 60% B for 9–18 min, increased linearly from 60% B to 100% B for 18–20 min, and remained at 100% B for 20–30 min. To avoid cross-contamination of plasma samples, we performed an ultra-pure water elution after each sample. All data were collected in ionization quadrupole time-of-flight mass spectrometry with positive (ESI+) and negative (ESI−) full scan modes. Mass spectrometry parameters were as follows: GAS1, 80; GAS2, 70; CUR, 40; TEM, 375 °C; and ISVF, 5500 V; a mass range of 50 to 1500; scan time of 0.3 s.

### 4.3. Data Processing and Statistical Analysis

Data files were converted to the mzXML format using Agilent MassHunter qualitative analysis software (version B.01.00, Agilent Technologies, Santa Clara, CA, USA) and processed using XCMS online (https://xcmsonline.scripps.edu, accessed on 8 June 2021), which performed feature detection, retention time correction, alignment, annotation, etc. Metabolic peaks detected less than 80% in all QC samples were discarded [54]. Identification of metabolites was carried out by matching *m*/*z* (Da) values against the Human Metabolome Database (www.hmdb.ca, accessed on 15 September 2021). Before statistical analysis, each metabolic peak in all subject samples was normalized based on QC samples for removing the unwanted analytical variations occurring intra- and inter-batches. And the plasma-abundant values of metabolites investigated were set to a log scale and auto-scaled (mean-centered and divided by the standard deviation of each variable) using MetaboAnalyst (https://www.metaboanalyst.ca, accessed on 10 November 2021).

Survival rate was calculated by the Kaplan–Meier method, log-rank tests were used to compare the survival difference between two or multiple groups, and multivariate Cox regression analyses were used to analyze the relationship between metabolites and the prognosis of GC, further calculating the hazard ratios (HRs) and its 95% confidence interval (CIs). Nomogram was constructed to visualize the results of the multivariate analysis. Surv_cutpoint (“survminer”, R package) was used to separate plasma metabolites into two groups, Lasso regression was used to screen and model the metabolites most associated with survival. After calculating the risk score for each patient according to the formula (Equation (1)), the X-tile software (version 3.6, Yale University, New Haven, CT, USA) was used to find the best cut-off value. The receiver operating characteristic (ROC) curves were used to evaluate the predictive performance of the models. The MetaboAnalyst platform was used for metabolite pathway analysis. SPSS 18.0 and R 4.0 software packages were used to complete the above analysis. All *p* values were based on the bilateral test, and the statistical test level was α = 0.05.

Y = β_1_ X_1_ +β_2_ X_2_ + … + β_8_ X_8_ (1)

where Y is the Risk score, X is the expression metabolite, and β is the coefficient.

## Figures and Tables

**Table 1:** Basic information about the research subjects.

		N	Kaplan–Meier		Cox Regression	
			5-Year of Survival Rate (%)	Log-Rank *p*	HR (95% CI)	*p*
Gender				0.617		
	male	162	37.49		1.00	
	female	56	37.50		0.906 (0.617–1.33)	0.615
Age				0.043 *		
	<65	45	51.11		1.00	
	≥65	173	33.97		1.588 (1.007–2.506)	0.047 *
TNM staging				<0.001 ***		
	early	41	87.80		1.00	
	middle	74	53.40		4.223 (1.771–10.072)	0.001 **
	late	103	5.83		24.457 (10.481–57.069)	<0.001 ***
Surgery				<0.001 ***		
	no	78	16.67		1.00	
	yes	140	49.06		0.345 (0.245–0.484)	<0.001 ***
Chemotherapy				0.007 **		
	no	102	30.39		1.00	
	yes	116	43.76		0.634 (0.454–0.885)	0.007 **
Radiotherapy				0.420		
	no	189	36.34		1.00	
	yes	29	44.83		0.809 (0.48–1.364)	0.427
Tumor location				0.184		
	Not in Cardiac	71	46.48		1.00	
	In Cardiac	147	33.10		1.287 (0.89–1.861)	0.179

HR, the hazard ratio; CI, the confidence interval; * *p* < 0.05, ** *p* < 0.01, *** *p* < 0.001.

## Data Availability

The data presented in this study are available on request from the corresponding author. The data are not publicly available due to restrictions of privacy.
